# Effects of kettlebell swing training on cardiorespiratory and metabolic demand to a simulated competition in young female artistic gymnasts

**DOI:** 10.1371/journal.pone.0283228

**Published:** 2023-04-24

**Authors:** Xavier Melo, Inês Arrais, João Luís Marôco, Pedro Neto Ribeiro, Sara Nabais, Raquel Coelho, Joana Reis, Vítor Angarten, Bo Fernhall, Helena Santa-Clara

**Affiliations:** 1 Centro de Investigação Interdisciplinar Egas Moniz (CiiEM), Egas Moniz School of Health and Science, Almada, Portugal; 2 Faculdade de Motricidade Humana–Universidade de Lisboa, CIPER–Centro Interdisciplinar de Estudo da Performance Humana, Lisboa, Portugal; 3 Ginásio Clube Português, Research & Development Department, Lisboa, Portugal; 4 Center for Sports Optimization (COD), Sporting Clube de Portugal, Lisbon, Portugal; 5 Exercise and Health Sciences Department, University of Massachusetts Boston, Boston, Massachusetts, United States of America; Universita degli Studi di Milano, ITALY

## Abstract

We examined the effects of adding a Kettlebell Swing training program (KB) to the regular skill-training protocol (REGULAR) on cardiorespiratory fitness, cardiorespiratory/metabolic demand, and recovery to a simulated competition of female artistic gymnastics. Nine gymnasts (13±2 years) had their REGULAR complemented with a 4-week kettlebell training (REGULAR+KB), consisting of 3 sessions/week of 12x30” swings x 30” rest with ¼ of their body weight, while 9 aged-matched gymnasts acted as a comparison group. Peak oxygen uptake ($\dot{V}O_{2ext}$) during routines was estimated from the O_2_ recovery curve using backward extrapolation and off-kinetics parameters were modeled through a mono-exponential function. Heart rate (HR) was monitored continuously and capillary blood lactate (BL_a_^-^) was measured before and after each routine (1^st^ and 3^rd^ min). Cardiorespiratory fitness ($\dot{V}O_{2\;\max}$) was evaluated using a ramp cycle ergometer test. A training-by-time interaction effect was observed for $\dot{V}O_{2\max}$ (*p* = 0.009) as increments were only observed after REGULAR+KB (M = 8.85, SD = 9.67 ml.kg.min^-1^). No training-by-time interactions were observed for *HR*_*peak*_ (*p =* 0.39), $\dot{V}O_{2\;ext}$ (*p* = 0.07), or *La*^*-*^_*post3 (*_*p =* 0.25*)*_,_ both training protocols reduced *HR*_*peak*_ (M = -12; SD = 11 b.min^-1^) and *BLa*^*-*^_*post1*_ (M = -0.70; SD = 1.29 mmol.L^-1^) during the simulated competition, but not relative $\dot{V}O_{2ext}$. No training-by-time interaction was observed for the off-transient $\dot{V}O_{2}$ time constant (*p* = 0.38). $\dot{V}O_{2}$ recovery was slower (M = 5; SD = 10 s) after both protocols. Both training protocols improved cardiorespiratory and metabolic demands and recovery kinetics to a simulated competition of female artistic gymnastics, although increases in cardiorespiratory fitness were only observed in REGULAR+KB.

## Introduction

Female artistic gymnastics is a highly technical and physiologically demanding sport consisting of four apparatus routines (vault, uneven bars, balance beam, and floor). Not surprisingly, artistic gymnastics is an early specialization sport, typically involving high training loads during the prepubertal years aimed at mastering both motor abilities (e.g., flexibility, strength, endurance) and skills relevant to performance [1]

The remarkable development of female artistic gymnastics seen over the past decade has influenced the Code of Points and judging, leading to a noticeable increase in the technical cardiorespiratory, and metabolic demands of the discipline [2]. In fact, peak blood lactate concentrations range from 4.7 to 10.5 mmol.L-1, peak heart rate (HRpeak), and oxygen uptakes reaching values of 174 to 203 beats.min-1, and 38.57 to 56.40 mL.kg-1.min-1, respectively, have been reported during the 5 to 90-s artistic gymnastics competitive routines. Therefore, high levels of physical fitness are required during artistic gymnastics routines in which anaerobic energy systems play a key role [3, 4]. The importance of the contribution of aerobic energy to the performance of competitive gymnastics artistic routines has also been recently acknowledged [3], refuting previous misconceptions among coaches that this pathway played a negligible role in short-duration exercises. In fact, equal energy contributions from the aerobic and anaerobic energy systems occur ~75-s after the onset of exercise [5]. Overall, these findings suggest that energy contributions, heart rate (HR), and blood lactate (BLa-) kinetics are apparatus-dependent [3, 6]. Artistic gymnastics routines lasting >30-s (i.e., floor routine) impose high cardiorespiratory demands. Therefore, artistic gymnastics cause high metabolic and cardiorespiratory stress that can hinder performance if recovery is not quick. Adaptations to exercise training significantly increase maximal oxygen uptake ($\dot{V}O_{2\;\max}$) and reduce the time required for $\dot{V}O_{2}$ to return to baseline after exercise [7], resulting in a faster homeostatic recovery of $\dot{V}O2$. However, little is known about time and energy-efficient training methods to improve these determinants in female artistic gymnastics.

Ballistic and/or ground-type lifting full-body exercises while supporting an external load using high muscle forces are potentially useful methods to increase strength, power, and endurance [8]. In fact, a variety of kettlebell protocols have been used in strength and conditioning in mixed martial arts [9], handball [10], shot put [11], sprinting [12], and soccer [13]. Therefore, kettlebell training has become part of elite training methods in sports with intermittent profiles, that rely on high contributions of anaerobic energy [14]. Given the metabolic similarities of these sports with artistic gymnastics, kettlebell exercise could be a suitable training method to increase the strength, power, endurance, and ultimately performance of artistic gymnasts. However, it remains unknown whether the efficacy of claims credited with kettlebell exercise is translated into female artistic gymnastics.

Therefore, this study aimed to examine the effects of adding a 4-week kettlebell swing training program to the regular skill-training protocol already performed by female artistic gymnasts (REGULAR+KB), on cardiorespiratory fitness, cardiorespiratory and metabolic demands, and recovery during simulated competition. We hypothesized that REGULAR+KB would induce superior gains in cardiorespiratory fitness in female artistic gymnastics, and faster recovery after routines compared to regular skill training alone (REGULAR).

## Methods

### Experimental approach to the problem

This study was designed as a quasi-experimental repeated measures intervention with two competitive simulations conducted before and after the intervention. We conducted this study during the in-season of young female artistic gymnastics with participants being assigned by age, space, and time convenience to REGULAR+KB or REGULAR. The experimental training intervention consisted of a 4-week REGULAR+KB, whereas the comparison group performed REGULAR. The two groups were led by the same strength, conditioning, and gymnastics coaches. Each pre- and post-intervention testing were completed in 2 visits, 48 h apart. Post-intervention tests were conducted 72 h after the final training session. On the first experimental day, gymnasts completed a cardiopulmonary exercise testing and body composition evaluation. On the second experimental day, gymnasts completed a four-routine (vault, uneven bars, balance beam, and floor) competitive simulation in randomized order (https://www.randomizer.org/) with at least a 10-min recovery between each routine. Gymnasts performed one at a time, in the same order, and were judged by international referees based on the following criteria: execution (E), difficulty (D), and total scores. BL_a_^-^ and Oxygen uptake ($\dot{V}O_{2}$) were collected before and following (1^st^ and 3^rd^ min for BL_a_^-^) each routine, whereas HR was monitored throughout the entire competition.

This study was conducted according to the Declaration of Helsinki and was approved by the Ethics Review Board of the Faculdade de Motricidade Humana–Universidade de Lisboa (12/2019).

### Subjects

Eighteen young female artistic gymnasts (12–17 years old, 1–4 Tanner stages) were sampled by convenience to participate in this study. This group was elite-oriented with training schedules up to 30 h per week over 8 weekly sessions and with competitive experience (approximately 3–7 years). All gymnasts were medically cleared and written parental consent and informed assent were obtained prior to each intervention.

### Procedures

#### Body composition assessment

Height was measured to the nearest 0.1 cm on a scale with an attached stadiometer (model 770, Seca; Hamburg, Germany), and waist circumference (WC), an estimate of subcutaneous and intra-abdominal (e.g., visceral) adipose tissue [15], was measured to the nearest 0.1 mm with an elastic metallic tape (Lufkin—W606PM, Vancouver, Canada) at the level of the iliac crests. Body mass (kg), fat mass (kg), and free fat mass (kg) parameters were obtained by a bioimpedance device (mBCA 515, SECA; Hamburgo, Germany) using four pairs of electrodes (eight electrodes in total) that allow the measurement of segmental impedance from a 100 μA current at frequencies between 1 and 1 000 kHz. Participants were assessed in a fasted state (>3h) and refraining from drinking alcohol, caffeine, and vigorous exercise in the 12h prior to the impedance measurement.

#### Maturity

Maturity was anonymously assessed with a validated self-assessment questionnaire with illustrations based on the 5 Tanner pubertal stages [16].

#### Cardiorespiratory fitness

Cardiorespiratory fitness was measured before and after intervention using a continuous ramp cycle ergometer (Monark 839 E Ergomedic; Monark, Vansbro, Sweden) protocol designed to achieve a peak effort of exercise in 8–10 min [17]. Work rate increased by 0.3 per kg of body weight per minute (W.kg^-1^.min^-1^). All gymnasts had work rate increases of 25 Watts/min. Pulmonary gases were continuously analyzed, with breath-by-breath gas exchange measurements, through a portable gas analyzer (K5, Cosmed, Rome, Italy). Data were analyzed by a single researcher in 10-s averages, and $\dot{V}O_{2\;\max}$ was defined as the highest 10-s value attained in the last minute of effort provided 2 of the following criteria are met: (1) Attaining ≥ 90% of age-predicted maximal HR; (2) Plateau in $\dot{V}O_{2}$ response despite increases in workload (<2.0 mL.kg^-1^.min^-1^); (3) Rating of perceived exertion ≥ 18 (6–20); (4) Respiratory exchange ratio ≥ 1.10; (5) unable to maintain a pedaling cadence above 70 revolutions.min^-1^; and (6) subjective judgment by the observer that the participant could no longer continue, even after encouragement [18]. The first ventilatory threshold (VT1) was defined as 1) the first increment in the ventilatory equivalent for O_2_ (VE/VO_2_), without any increase in the ventilatory equivalent of CO_2_ (VE/VCO2), and 2) the first increase in the expiratory fraction of O2. The second ventilatory threshold (VT2) was defined as 1) the first increment in the ventilatory equivalent for CO_2_ (VE/VCO_2_), and 2) the first decrease in the CO_2_ expiratory fraction. Before each test, both O_2_ and CO_2_ analyzers were calibrated using ambient air and standard calibration gases of known concentration (16.7% O_2_ and 5.7% CO_2_). The calibration of the turbine flowmeter of the K5 was performed using a 3-l syringe, according to the manufacturer’s instructions. Heart rate was continuously monitored (Garmin, US).

#### Physiological demands evaluations during the simulated competitive routines oxygen uptake and heart rate

Pulmonary gases were analyzed in the standing position before (1-min) and immediately following each routine (3-min), with a breath-by-breath portable gas analyzer (K5, Cosmed, Rome, Italy). This methodological approach precluded the recording of the ventilatory parameters during the routine but permitted the assessment of the highest possible values within < 5-s after completion. Peak oxygen uptake ($\dot{V}O_{2\;ext}$) during routines was estimated by backward extrapolation of the VO_2_ recovery curve to time zero [19], assumed as the moment when the gymnasts finished their schemes. HR was monitored using chest straps during the simulated competitive events (Garmin, USA).

Despite the suggestion that maximal exercise off-transient is better characterized by a bi-exponential model for either fast or slow $\dot{V}O_{2}$ components, the model that best fits the data should be used. Thus, $\dot{V}O_{2}$ recovery was modeled with a mono-exponential based on the excess post-exercise oxygen consumption of the fast component using the following equations [20]:

$${\dot{V}O}_{2(t)}={\dot{V}O}_{2(pre)}+A_{p}\left(e^{-(\frac{t-\delta }{\tau_{p}})}\right)$$

$${EPOC}_{FAST}=A_{p}\times \tau_{p}$$

Where $\dot{V}O_{2(t)}$ is the value of $\dot{V}O_{2}$ at a determined time (mL.min^-1^) $\dot{V}O_{2(pre)}$ is the pre-routine value of $\dot{V}O_{2}$ (mL.min^-1^), *A*_p_ is the amplitude of $\dot{V}O_{2}$ (mL.min^-1^), *δ* is the time delay and τ_p_ is the time constant. The overall goodness of the fit for the off-transient models was good for the $\dot{V}O_{2}$ kinetics models for the vault (R^2^ = 0.75), uneven bars (R^2^ = 0.77), balance beam (R^2^ = 0.80) and floor (R^2^ = 0.83) routines.

#### Capillary blood lactate

Capillary blood lactate was collected from the ear lobe using a portable lactate analyzer (Lactate Pro2, Arkray, Japan) before (within the previous min) and immediately following each routine (1^st^ and 3^rd^ min). Before each collection the earlobe was cleaned with an alcohol swab, pricked and the first blood drop was wiped with a tissue, then the earlobe was squeezed, and the subsequent blood drop was used.

### Training interventions

#### Kettlebell swing

The REGULAR+KB was a 4-week strength/power training program involving 3 sessions/week of Kettlebell Swing, with 48h in-between was added and performed before to the regular female artistic gymnastics training session, consisting of 12 rounds of 30-s swing exercise separated by 30-s of rest. Gymnasts were instructed to perform as many swings as possible during each round using a Kettlebell weighting ¼ of their body weight [8, 21–23], with the technique previously outlined [8]. The number of swings performed during each round was recorded and served as vocal encouragement throughout each 12-minute bout. The protocol was led by a strength and conditioning coach with a specialization in kettlebell training. During this period, gymnasts did not perform any other resistance exercise besides their REGULAR in their common gymnastics room and with the same coaching staff.

#### Regular skill-training protocol (REGULAR)

The REGULAR was performed at the early hour of each training session and consisted of 1 set of 11 exercises, with a different number of repetitions according to the physical demands of each exercise. These included planks at different heights, walking weights, handstands on the floor or balance beam, rope jumps on the balance beam, various jumps at different heights, pull-ups on uneven bars, front-flips, and back-flips. Gymnasts have performed REGULAR or slight variations since the beginning of their sports career in this modality.

#### Standard nutritional plan

A 1-day dietary plan was given to each gymnast before the field test day at both pre-and post-intervention assessments, in conformity with the nutritional plan elaborated earlier in the season by the nutritionist. Aspects such as food portions, the number of daily meals, and supplements were described in the nutritional plan, specific to each participant and following the requirements of the gymnasts. Gymnasts were asked to refrain from smoking cigarettes or drinking alcohol or coffee in the 24 hours prior to the assessment day.

### Statistical analysis

Based on an effect size of 0.57, a priori power analysis (GPower Version 3.1.9.3) for mixed ANOVA suggested a total of 18 participants are required to detect a previously reported $\dot{V}O_{2\;\max}$ mean difference of 2.3 mL.kg.min^-1^ (SD = 2.0) over time and training protocols (α = 0.05, 1−β = 0.95) [24].

Data are presented as mean (SD) unless otherwise indicated. The normality and homoscedasticity assumptions were verified with the Shapiro-Wilk and Levene tests, respectively, and by plot inspection. Independent samples t-tests were used to compare the baseline characteristics of the participants.

The changes in the dependent variables in pre-and-post exercise interventions and competitive simulations were examined using linear mixed models fitted with restricted maximum likelihood and applying Satterthwaite’s method for approximating degrees of freedom for the F test from the R lmerTest package [25]. The fixed effects were defined as time, exercise training protocols, and routines, and the random intercept was defined for each participant. Partial eta squares (*η*^*2*^) were calculated for each main effect and interactions of interest (i.e., training-by-time, routine-by-time) using the R sjstats package [26] and interpreted by applying the benchmarks suggested by Cohen [27] defining small (*η*^*2*^ < 0.05), medium (*η*^*2*^ < 0.25), and large (*η*^*2*^ > 0.25) effect sizes. Main outcomes during simulation events were covaried for routines starting order and maturation status. The changes induced by exercise training interventions in $\dot{V}O_{2\;\max}$ were adjusted for baseline values. Post-hoc comparisons using Tukey’s HSD test were applied with the R emmeans package [28] in the presence of significant differences in main effects and interactions. All statistical analyses were conducted using R, version 4.0.1 [29], with a significant level (α) of < 0.05.

## Results

### Characteristics of the participants

**Fig 1:**
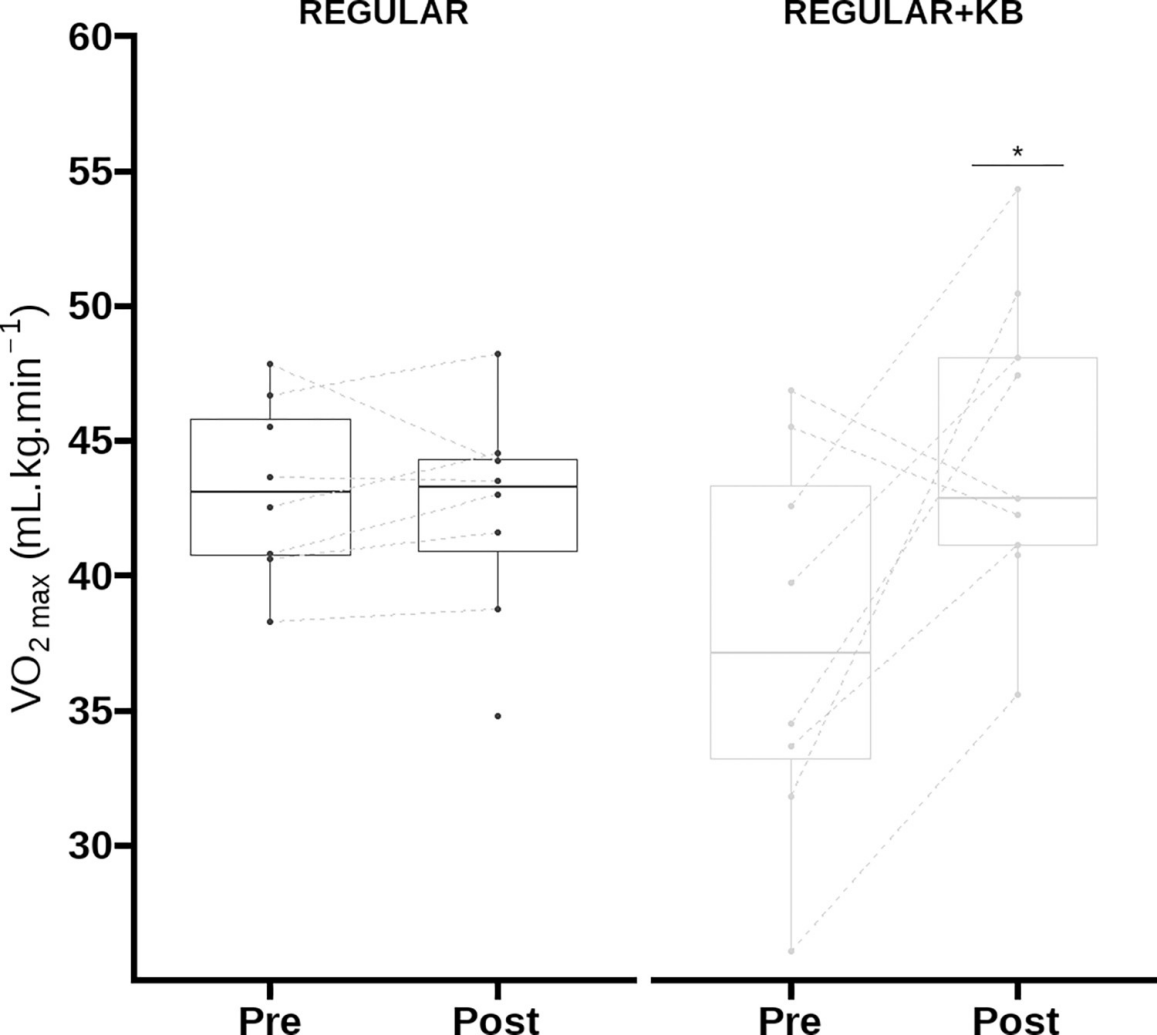
Effects of REGULAR and REGULAR+KB in selected cardiorespiratory fitness indices derived from the cardiopulmonary exercise test. Abbreviations: REGULAR, skill training; REGULAR+KB, regular skill training protocol + Kettlebell training; $\dot{V}O_{2\max}$; maximum oxygen uptake; $\dot{V}O_{2}$ at the second ventilatory threshold. *Indicates difference from values before the interventions (p < 0.05).

The characteristics of the participants did not differ between groups, except for the relative $\dot{V}O_{2\;\max}$ that was higher in REGULAR than in REGULAR+KB (Table 1, Fig 1).

**Table 1:** The characteristics of the participants.

Characteristics	REGULAR (n = 9)	REGULAR+KB (n = 9)	*p*-value
Age (years)	14 (2)	13 (2)	0.5
Height (m)	1.54 (0.1)	1.53 (0.1)	0.8
Weight (kg)	48.0 (8.0)	44.0 (10.1)	0.4
Body mass index (kg.m^-2^)	20.1 (1.6)	18.5 (2.4)	0.14
Waist circumference (cm)	66 (3)	65 (6)	0.8
% Body fat mass (%)	13.3 (4.1)	9.1 (6.5)	0.13
Body fat-free mass (kg)	41.4 (6.2)	39.3 (7.3)	0.5
$\dot{V}O_{2\;\max}$ (L.min^-1^)	1.84 (0.78)	1.53 (0.36)	0.3
$\dot{V}O_{2\;\max}$ (mL.kg^-1^.min^-1^)	43.25 (3.30)	36.00 (8.45)	0.04

Data presented as mean (SD), ^1^Statistical tests performed: independent Welsch t-test, Abbreviations: REGULAR, regular training; REGULAR+KB, regular skill training protocol + Kettlebell training; $\dot{V}O_{2\;\max}$ maximal oxygen uptake

### Cardiorespiratory fitness and body composition

Training-by-time interaction effects were observed for relative $\dot{V}O_{2\;\max}$ [F (1,15) = 8.87, *p* = 0.009, η^2^ = 0.37], and relative $\dot{V}O_{2}$ at VT2 [F (1,15) = 8.22, *p* = 0.01, η^2^ = 0.35; Fig 1; S1 File], suggesting that REGULAR+KB increased $\dot{V}O_{2\;\max}$ (d = 8.85; 95% CI: 2.78 to 14.9 mL.kg^-1^.min^-1^, *p* < 0.001) and the $\dot{V}O_{2}$ at VT2 (d = 9.84; 95% CI: 2.73 to 16.94 mL.kg^-1^.min^-1^, *p* < 0.001) whereas REGULAR did not. No training-by-time interactions or main effects of time or training were observed for fat mass, and *HR*_*max*_ (S1 File). Adjustments for maturation status and baseline values of relative $\dot{V}O_{2\;\max}$ and relative $\dot{V}O_{2}$ at VT2 did not alter the results.

### Cardiorespiratory and metabolic demands of routines

#### Pre-routines

No training-by-time interaction effects were observed for relative $\dot{V}O_{2\;\mathrm{pre}}$, [F (1, 107) = 2.897, *p* = 0.09, η^2^ = 0.03], BLa^*-*^_*pre*_ [F (1, 108) = 0.004, *p* = 0.95, η^2^ = 0] or HR_pre_ [F (1, 108) = 3.06, *p* = 0.08, η^2^ = 0.03]. A routine-by-time interaction effect was observed for relative $\dot{V}O_{2\;pre}$ [F (3,103) = 3.11, *p* = 0.03, η^2^ = 0.08], as it was reduced before the floor routine following both exercise training protocols (d = - 2.99; 95% CI: -5.04 to—0.95 mL.kg^-1^.min^-1^, *p* < 0.001; Table 3). No significant differences were observed in $\dot{V}O_{2\;pre}$ in the remaining routines. Still, a main effect of time (F (1,107) = 19.64, *p* < 0.001, η^2^ = 0.16) and routine [F (3,103) = 33.77, *p* < 0.001, η^2^ = 0.50] was observed for relative $\dot{V}O_{2\;pre}$, suggesting that both exercise training protocols reduced relative $\dot{V}O_{2\;pre}$ (d = - 1.45; 95% CI: - 2.1 to -0.8 mL.kg^-1^.min^-1^, *p* < 0.001). The highest relative $\dot{V}O_{2\;pre}$ was observed before the floor routine, whereas the lowest was observed before the vault routine (d = 4.58; 95% CI: 3.38 to 5.77 mL.kg^-1^.min^-1^, *p* < 0.001). The uneven bars and balance beam routines showed an identical $\dot{V}O_{2\;pre}$. Adjustments for maturation status and routine order did not alter the results. *BLa*^*-*^_*pre*_ (d = - 0.40; 95% CI: -0.59 to -0.21 mmol.L^-1^, *p* < 0.001) and *HR*_*pre*_ (d = - 7; 95% CI: -9 to -4 b.min^-1^, *p* < 0.001) decreased following both exercise training protocols.

#### During routines

No training-by-time interaction effects were observed for *HR*_*peak*_ [F (1,107) = 0.75, *p* = 0.39, η^2^ = 0] and relative $\dot{V}O_{2\;ext}$ [F (1,108) = 3.43, *p* = 0.07, η^2^ = 0.02, Table 3]. Still, a main effect of time [F (1,107) = 46.25, *p* < 0.001, η^2^ = 0.30] and routine [F (1,103) = 123.80, *p* < 0.001, η^2^ = 0.78] was observed for *HR*_*peak*_, suggesting that both exercise training protocols reduced *HR*_*peak*_ during routines (d = -12; 95% CI: -15 to -8 b.min^-1^, *p* < 0.001). The highest *HR*_*peak*_ was attained during the floor whilst the lowest was in the vault routine (d = 42; 95% CI: 36 to 48 b.min^-1^, *p* < 0.001). *HR*_*peak*_ was similar between the balance beam and uneven bars routines. The highest relative $\dot{V}O_{2\;ext}$ was achieved during the floor and uneven bars routines, while the lowest was in the vault routine (S3 File). Additionally, only the vault routine showed a %HRmax below 90% (Table 2). Adjustments for maturation status and routine order did not change these results. There were no differences in the routine scores between groups both before and after exercise training protocols (*p* = 0.46) (S2 File).

**Table 2:** Effects of exercise training protocols on oxygen uptake, blood lactate, and heart rate before and during female artistic gymnastics routines.

	REGULAR				REGULAR+KB				*Time*	*Routine*	*Training*	*Interaction*
	Vault	Bars	Beam	Floor	Vault	Bars	Beam	Floor	p (η^2^)	p (η^2^)	p (η^2^)	p (η^2^)
$\dot{V}O_{2\;pre}$ (mL.kg. ^-1^min^1^)												Routine*time
Pre	4.35 (1.62) #δ¥	6.68 (2.23)	7.73 (1.39)	10.09 (2.99) ⱡ δ¥	8.93 (3.25) #δ¥	11.12 (2.66)	11.57 (2.04)	14.71 (3.56) ⱡ δ¥	**<0.001** (0.16)	**<0.001** (0.50)	**0.002** (0.67)	**0.03** (0.08)
Post	4.87 (1.78) #δ¥	6.32 (0.96)	5.73 (1.08)	8.26 (1.63)*	7.054 (2.3) #δ⸷	10.43 (1.37)	10.26 (2.46)	10.5 (2.41)*				
*BLa*^*-*^_*pre*_ (mmol.L^-1^)												
Pre	2.98 (0.93) δ#	3.36 (1.107) δ#	2.27 (1.11)	2.08 (0.70)	2.58 (0.86)δ#	2.31 (0.50)δ#	1.74 (0.40)	1.46 (0.31)	**<0.001** (0.14)	**<0.001** (0.45)	**<0.001** (0.45)	
Post	3.00 (0.42)*	2.78 (0.60)*	1.88 (0.49)*	1.81 (0.29)*	2.04 (0.67)*	1.75 (0.53)*	1.44 (0.42)*	1.23 (0.29)*				
HR _pre_(b.min^-1^)												
Pre	109 (17)	114 (16)	114 (9)	111 (16)	115 (19)	110 (17)	118 (13)	114 (20)	**<0.001** (0.19)	0.06 (0.07)	0.49 (0.03)	
Post	107 (9)*	107 (7)*	107 (9)*	103 (5)*	108 (16)*	108 (16)*	116 (16)*	109 (21)*				
*HR_peak_* (b.min^-1^)												Training*routine
Pre	143 (14)*	180 (7)	181 (7)	190 (3)	157 (19)	177 (12)	180 (10)	188 (7)	**<0.001** (0.30)	**<0.001** (0.78)	0.32 (0.06)	**0.03** (0.08)
Post	128 (13)	163 (8)	173 (9)	180 (7)	137 (16)	172 (12)	177 (9)	176 (19)				
*HR_max_* (%)												
Pre	82 (11) #δ¥	101 (5)	102 (6)	89 (40)	90 (11) #δ⸷	102 (4)	103 (3)	108 (5)	0.05 (0.03)	**<0.001** (0.41)	**0.04** (0.23)	
Post	72 (9) #δ¥	93 (8)	99 (6)	103 (5)	78 (8) #δ⸷	98 (6)	101 (3)	100 (12)				
$\dot{V}O_{2\mathrm{ext}}$ (mL.kg.^-1^min^-1^)												
Pre	22.88 (6.77) #¥	38.37 (4.36)	29.02 (4.98)#¥	31.87 (14.49)	35.42 (10.47)#¥	43.59 (9.43)	40.01 (10.08) #¥	46.91 (10.76)	0.82 (0)	**<0.001** (0.28)	**<0.001** (0.49)	
Post	25.94 (6.17)	33.4 (5.03)	29.86 (2.70)	41.58 (6.28)	38.11 (11.06)	42.82 (7.12)	37.72 (5.15)	39.14 (6.78)				
$\dot{V}O_{2ext}$(%)												Training*time
Pre	53 (17)	89 (12)	68 (14)	64 (40)	103 (36) # δ ⱡ	128 (40) # δ ⱡ	116 (37) #¥ ⱡ	135 (31) ¥ⱡ δ	**<0.001** (0.13)	0.08 (0.05)	**<0.001** (0.53)	**0.006** (0.06)
Post	50 (23)	66 (27)	64 (27)	89 (39)	85 (23)*	96 (10)*	85 (13)*	87 (10)*				

Data presented as mean (SD); Abbreviations: REGULAR, regular skill training; REGULAR+KB, regular skill training protocol + Kettlebel training; HR_pre_, resting heart rate; HR_peak_, peak heart rate during the routines, $\dot{V}O_{2\;pre}$; oxygen uptake at rest; *BLa*^*-*^_*pre*_, resting lactate before each routine, $\dot{V}O_{2\;ext}$ extrapolated maximal oxygen uptake during the routines. Post-hoc comparisons were performed for the routine-by-time interaction for each group, the protocol-by-routine interaction for the main effect of routine, and for the main effects of routine and time. Only significant interactions are reported. * Indicates a difference from pre (*p* < 0.01). # Indicates a difference from the floor routine (*p* < 0.01), ⱡ Indicates a difference from the vault routine (*p* < 0.01), δ Indicates difference from balance beam routine (*p* < 0.01), ¥ Indicates a difference from uneven bars routine (*p* < 0.01).

**Table 3:** Effects of exercise training protocols on oxygen uptake and blood lactate after the artistic gymnastics routines.

	REGULAR				REGULAR+KB				*Time*	*Routine*	*Training*	*Interaction*
	Vault	Bars	Beam	Floor	Vault	Bars	Beam	Floor	p(η^2^)	p (η^2^)	p(η^2^)	p(η^2^)
τ(s)												
Pre	47 (19)	50 (7)	38 (8)	50 (6)	49 (10)	48 (10)	45 (8)	49 (7)	**0.003** (0.08)	**0.003** (0.13)	0.78 (0)	
Post	56 (17)	55 (11)	48 (8)	57 (9)	57 (15)	49 (10)	48 (5)	51 (12)				
A_p_ (mL.min^-1^)												
Pre	1009 (342) #δ¥	1574 (477)	1475 (385)	1583 (510)	1195 (389) #δ¥	1624 (389)	1390 (322)	1603 (157)	0.12 (0.02)	**<0.001** (0.31)	0.87 (0)	
Post	1247 (356)	1532 (353)	1407 (267)	1811 (462)	1294 (184)	1497 (191)	1473 (285)	1435 (255)				
*BLa*_*post1*_(mmol.L^-1^)												Routine*time
Pre	2.90 (0.89)	4.46 (1.70) δ¥	3.08 (0.78)	5.95 (2.35) ¥ ⱡ δ	2.53 (0.58)	3.68 (0.79) ⱡ δ	2.31 (0.47)	6.41 (3.20) ¥ⱡ δ	**<0.001** (0.14)	**<0.00**1 (0.57)	0.64 (0.01)	**0.03** (0.08)
Post	2.59 (0.52)	3.35 (0.24)	2.42 (0.45)	4.27 (0.87)*¥ⱡ δ	2.28 (0.65)	3.09 (0.81)	2.43 (0.62)	4.70 (1.44) *¥ⱡ δ				
*BLa*^*-*^_*post3*_ (mmol.L^-1^)												
Pre	2.80 (0.74) ⱡ δ	4.11 (1.27)	2.60 (0.61)	6.42 (2.28) ⸷ⱡ δ	2.30 (0.48) ⱡ δ	3.47 (0.60)	2.32 (0.83)	5.63 (1.07) ¥ⱡ δ	**<0.00**1 (0.18)	**<0.001** (0.76)	0.23 (0.09)	
Post	2.41 (0.57)*	3.39 (0.54)*	2.25 (0.48)*	4.68 (0.97)*	1.93 (0.40)*	2.84 (1.12)*	2.02 (0.67)*	4.90 (1.47)*				
HRR1 (b.min^-1^)												Routine*time
Pre	30 (10)	48 (18)	35 (12)	41 (14)	41 (24)	45 (15)	38 (9)	42 (17)	0.08 (0.03)	**<0.001** (0.27)	0.5 (0.03)	**<0.001** (0.22)
Post	13 (11) *#δ⸷	35 (16)	51(10)*	38 (13)	23 (8) *#δ¥	41 (16)	49 (14)*	41 (36)				
HRR3 (b.min^-1^)												Routine*time
Pre	35 (10) #¥	48 (18)	51 (10) #¥	76 (16)	47 (20) #¥	45 (15)	52 (11) #¥	70 (12)	0.36 (0)	**<0.001** (0.66)	0.87 (0)	**<0.001** (0.25)
Post	21 (10) *#δ¥	56 (11)	66 (6)	73 (13)	30 (9) *#δ¥	62 (15)	67 (10)	65 (30)				

Data presented as mean (SD); Abbreviations: REGULAR, regular skill training; REGULAR+KB, regular skill training protocol + Kettlebel training; *τ_p_*, time constant of the $\dot{V}O_{2}$ in off-kinetics analysis; A_p,_ amplitude of the $\dot{V}O_{2}$ in off-kinetics analysis; *BLa*^*-*^_*post1*_ and *BLa*^*-*^_*post3*,_ lactate 1 and 3 minutes following each routine, respectively; *HRR1* and *HRR3*_,_ heart rate 1 and 3 minutes following each routine. Post-hoc comparisons were performed for protocol-by-time and routine-by-time interactions for each group, and for the main effects of time and routine. Only significant interactions are reported. * Indicates a difference from pre (*p* < 0.01). # Indicates a difference from the floor routine (*p* < 0.01), ⱡ Indicates difference from the vault routine (p < 0.01), δ Indicates difference from balance beam routine (*p* < 0.01), ¥ Indicates a difference from uneven bars routine (*p* < 0.01).

#### Recovery

**Fig 2:**
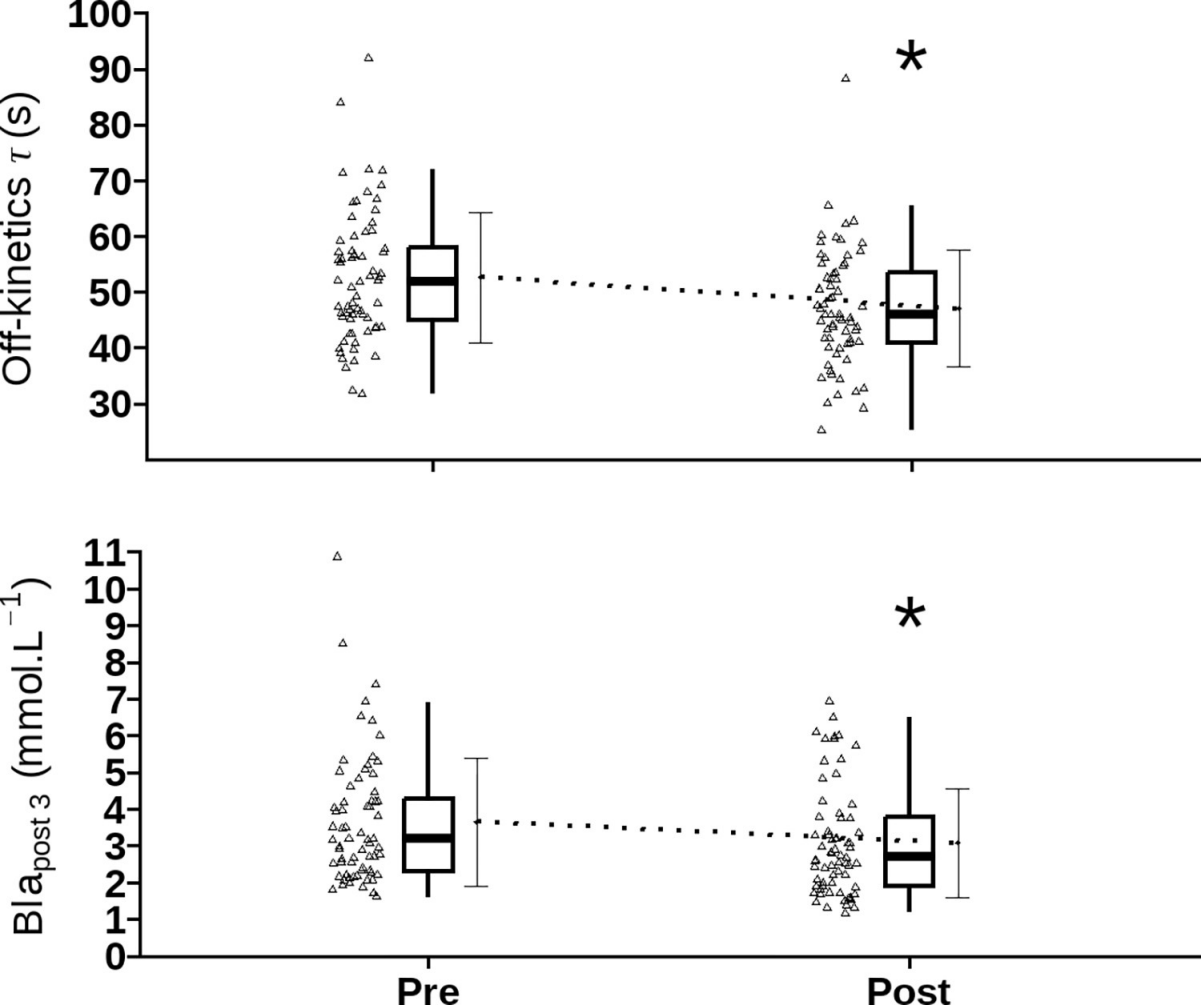
Effects of exercise training protocols on selected indices of $\dot{V}O_{2}$ off-kinetics and blood lactate during recovery from female artistic gymnastics routines. Abbreviations: REGULAR, skill training; REGULAR+KB, regular skill training protocol + Kettlebell training; $\dot{V}O_{2}$, oxygen uptake; *BLa*^*-*^_*post3*,_ blood lactate 3 minute following each routine. *Indicates significant difference from values before interventions (p < 0.05).

No training-by-time interaction effects were observed for relative *BLa*^*-*^_*post1*,_[F (1, 108) = 1.34, *p* = 0.25, η^2^ = 0.01], *BLa*^*-*^_*post3*_ [F (1, 108) = 1.87, *p* = 0.17, η^2^ = 0.02]. A routine-by-time interaction effect was observed for *BLa*^*-*^_*post1*_, suggesting that both exercise training protocols reduced *BLa*^*-*^_*post1*_ following the floor routine (d = -1.76; 95% CI: -2.95 to -0.56 mmol.L^-1^, *p* < 0.001) and uneven bars (d = -0.91, 95% CI: -1.68 to -0.15, p = 0.02) routines. The results also show that *BLa*^*-*^_*post3*_ decreased following exercise training (d = -0.70; 95% CI: -0.98 to -0.42 mmol.L^-1^, *p* < 0.001; Fig 2). *BLa*^*-*^_*post1*_ and *BLa*^*-*^_*post3*_ reached the highest values following the floor routine, while the lowest values were observed following the vault and balance beam routines (*BLa*^*-*^_*post1*,_ d = 2.77; 95% CI: 2.06 to 3.48 mmol.L^-1^, *p* < 0.001; *BLa*^*-*^_*post3*,_ d = 3.11; 95% CI: 2.60 to 3.62 mmol.L^-1^, *p* < 0.001 (S3 File). There was no training-by-time interaction effect for τ_p [_F (1, 107) = 0.78, *p* = 0.38, η^2^ = 0]. Both exercise training protocols increased the off transient τ_p_ of $\dot{V}O_{2}$ kinetics after routine cessation (d = 5; 95% CI: 2 to 8 s, *p* = 0.003; Fig 2. *A*_*p*_ was lowest in the vault routine compared to the remaining routines, where τ_p_ was lowest in the beam routine (Table 3, S3 File). The lowest *HRR1* and *HRR3* were observed following the vault routine compared to the remaining routines (S3 File, Table 3).

## Discussion

The main findings of this study were that REGULAR+KB did not add to the improvements in the cardiorespiratory and metabolic demands and recovery to a simulated female artistic gymnasts’ competition when compared to REGULAR. Both REGULAR+KB and REGULAR reduced the cardiorespiratory (*HR*_*peak*_) and metabolic (*BLa*^*-*^_*post1*_*)* demands of artistic gymnastics routines and slowed $\dot{V}O_{2}$ off-transient kinetics to facilitate PCr resynthesis between routines. Despite an increase in cardiorespiratory fitness noted only after KB, this did not translate to a reduction in cardiorespiratory and metabolic demand or to faster recovery between artistic gymnastics routines compared to regular training.

### Effects of exercise training interventions

REGULAR+KB was designed to address the recommendation by Marina and Rodríguez [3] for specialized cardiorespiratory fitness training [24], to facilitate recovery and comply with the increasingly complex and difficult profile of modern female gymnasts. In the present study, REGULAR+KB training led to an increase in $\dot{V}O_{2\;\max}$ and $\dot{V}O_{2}$ at VT2 during a progressive exercise test, whereas ST did not. Surprisingly, REGULAR+KB did not improve recovery more than REGULAR or reduce cardiorespiratory and metabolic demands, as evidenced by similar reductions in *HR*_*peak*_, *BLa*^*-*^_*post1*,_ and *BLa*^*-*^_*post3*_. Possible explanations may relate to the selected load [30, 31], the swing cadence [32], the duration [33] or, the lack of specificity of the REGULAR+KB protocol. We set the KB load at ¼ of the body mass criteria as suggested by previous work [21, 23, 34]. Wesley and Kivi [32] also reported that significant increases were seen in HR, *BLa*^*-*^, and perceived exertion for each 4 kg increase in KB load until ¼ body mass. However, Fung and Shore [30] recommended using less than 13% of body mass if the aim is to stress the aerobic system. Still, there is no consensus on the adequate load and cadence for a KB protocol. Swing cadence may also influence physiological responses as when swing cadence is increased to 48 swings per min, users are forced to actively pull the KB down during the backswing to keep pace, emphasizing an ‘overspeed eccentric’ action during the backswing. This increased pace results in significant increases in HR (14.7%), *BLa*^*-*^ (83%), and RPE (27%) compared to 40 swings per minute [35]. Furthermore, the effects of the exercise training protocols were measured only before and after 4 weeks, which may not have detected subtle changes in *HR*_*peak*_, *BLa*^*-*^_*post1*,_ and *BLa-post3*, or those that occurred outside of this initial time frame [7]. Finally, the REGULAR+KB protocol may not have met the specific requirements of gymnastics. Artistic gymnastics enlists highly skilled routines, performed in apparatus with significant levels of balance and isometric control of body mass that may not be reflected in a KB swing.

There is little evidence on the effects of exercise training on the off-transient $\dot{V}O_{2}$ kinetics. A few studies suggest that endurance exercise promotes faster off-transient $\dot{V}O_{2}$ kinetics leading to improvements in endurance performance [36, 37]. This is mainly due to faster on-transient kinetics, sustaining both a reduction in O_2_ debt and accumulation of fatigue-associated metabolites (e.g., H^+^, Pi, ADP). On the contrary, our results showed that both exercise training protocols slowed $\dot{V}O_{2}$ recovery in gymnasts. This finding should be interpreted considering the distinct metabolic demands of artistic gymnastics compared to endurance sports, as the energy substrate utilization during exercise is a determinant of the off-transient $\dot{V}O_{2}$ kinetics response. For example, Billat et al. [38] have shown that 4 weeks of endurance training reduced PCr depletion during running until exhaustion, accelerating the rapid phase of off transient $\dot{V}O_{2}$ kinetics. However, we observed that both exercise training protocols slowed off-transient $\dot{V}O_{2}$ kinetics after artistic gymnastics routines, suggesting a faster PCr resynthesis to compensate for the likely increase in phosphagen energy system contributions during the simulated competition routines. In fact, this tight coupling and inverse association between off-transient PCr and $\dot{V}O_{2}$ kinetics has been demonstrated in computational models [39, 40], although it remains to be replicated *in vivo* experiments. Even if we did not estimate τ_p_ for fast and slow phases of the $\dot{V}O_{2}$ recovery separately, the slow phase doubtfully accounts for the slower off-transient kinetics in our gymnasts, given that Bla^-^ was reduced following both exercise training protocols. Further, it appears that blood lactate accumulation *per se* is not accountable for the slower off-transient $\dot{V}O_{2}$ kinetics [38, 41]. Importantly, our data were best fitted by a mono-exponential model likely due to the asymmetry between on-and-off $\dot{V}O_{2}$ kinetics triggered by the near-maximal exercise intensity as reported by some [38], but not all [42, 43]. Furthermore, it is unlikely that the slower off-transient $\dot{V}O_{2}$ kinetics of female artistic gymnasts after training could be attributed to baseline differences compared to endurance athletes since we observed similar off-transient τ_p_ to those of running, cycling, and rowing (_~_ 50-s) athletes [42, 43]. This slower recovery of VO2 and the suggestion of faster PCr resynthesis after routine cessation induced by both exercise training protocols could be the key to improving artistic gymnastics performance, as the phosphagen system allows for well-known explosive gymnastic skills.

### Cardiorespiratory and metabolic demands of routines in female artistic gymnastics

In this study, female artistic gymnasts achieved the highest concentrations of *Bla*^*-*^_*post1*_ and *Bla*^*-*^_*post3*_ in the floor and uneven bars routines, which is consistent with a previous finding suggesting that the glycolytic anaerobic pathway plays a major role [3, 6]. The highest demand for oxygen delivery was observed in the floor, uneven bars, and balance beam routines, reaching up to 90% HR_max_ achieved during the cycle ergometer test, which aligns with previous results [3, 6].

The balance beam is considered by many artistic gymnasts the most difficult, stressful, and dangerous apparatus [3, 44, 45]. Adding to the fact that this routine requires prolonged isometric contractions [45], these observations may partially explain our findings. The vault routine showed the lowest HR_peak_, *Bla*^*-*^_*post1*,_ *Bla-post3*, and A_p_ among the routines, which is probably explained by its shorter duration and higher energetic contributions from the phosphagen system [5]. The slower off-transient $\dot{V}O_{2}$ kinetics (highest τ_p_) observed in the vault routine further supports its dependence on the phosphagen system. In fact, the PCr resynthesis occurs during the rapid phase of $\dot{V}O_{2}$ recovery and is inversely related to the off-transient $\dot{V}O_{2}$ kinetics, so that a slower recovery is associated with a higher level of PCr hydrolysis during exercise [37].

In the present study, the $\dot{V}O_{2}$, was higher in the floor routine, as previously reported by Marina and Rodríguez [3], followed by the uneven bars. These findings were further supported by the higher A_p_ observed in these routines. This reinforces previous observations [3, 6] that the cardiorespiratory and metabolic demands are both apparatus and routine-duration dependent, but also that the ATP resynthesis from anaerobic glycolysis is still predominant in female artistic gymnastics, as evidenced by the *BLa*^*-*^ concentrations following the routines.

### Importance of cardiorespiratory fitness in female artistic gymnastics

Research on female artistic gymnastics has reported a range of $\dot{V}O_{2\;\max}$ between 50 to 56 mL.kg^-1^.min^-1^ from running maximal exercise testing and that high $\dot{V}O_{2\;\max}$ would be key to accelerate recovery [3, 6]. However, the results of this study contradict these early findings as not only $\dot{V}O_{2\;\max}$ was lower in our gymnasts (36 to 43 mL.kg^-1^.min^-1^), as the increases observed following KB did not translate into faster $\dot{V}O_{2}$ recoveries after routines. First, the lower $\dot{V}O_{2\;\max}$ of our gymnasts is likely explained by the selected exercise testing modality, as cycling underestimates $\dot{V}O_{2\;\max}$ by 10 to 15% when compared to running tests [46]. This may also contribute to the lower $\dot{V}O_{2\;\max}$ of our gymnasts compared to other anaerobic sports such as sprint running and weightlifting [47], or wrestling [48], but not the higher when compared to rhythmic gymnasts [49]. Still, this is not a universal finding [50]. Second, our results suggest that increases in $\dot{V}O_{2\;\max}$ observed with KB do not translate to faster $\dot{V}O_{2}$ off-kinetics as they do for on-kinetics (i.e., inverse association between on transient τ_p_ and $\dot{V}O_{2\max}$) [37, 51]. This dissociation of $\dot{V}O_{2\;\max}$ with $\dot{V}O_{2}$ off-transient kinetics likely results from the mainly anaerobic energy profile of artistic gymnastics. Together, our findings shed doubt on the importance of high cardiorespiratory fitness to improve recovery between routines in artistic gymnastics, likely supporting conceptions coaches have that this pathway plays a negligible role in short-duration exercises.

### Limitations

This study is not without limitations. First, we used a convenience sample that may have lacked statistical power to support our results. Cardiorespiratory fitness was determined using a cycle ergometer that may not reflect the demands of artistic gymnastics. Second, artistic gymnasts have an exhaustive training schedule daily that focuses on almost every skeletal muscle group. The fact that REGULAR+KB only used the swing movement may have hampered our results. Indeed, the horizontal component of the movement can be seen in the floor, vault, and balance beam, but not in the uneven bars, which was the second most demanding routine for female gymnasts in this study. In addition, the REGULAR+KB had a higher training load compared to REGULAR, which might contribute to training load-dependent changes in our main outcomes. However, this is unlikely as both training protocols similarly improved the demand and recovery of cardiorespiratory and metabolic outcomes, and ultimately suggest that the additional workload was useless. Finally, the swing cadence was not controlled in the present study. This could have improved standardization throughout the protocol, although these gymnasts are familiarized with all-out efforts.

In conclusion, REGULAR+KB training did not add to the improvements in the cardiorespiratory and metabolic demands and recovery kinetics to a simulated female artistic gymnastics competition compared to REGULAR, despite the increases observed in $\dot{V}O_{2\;\max}$ after REGULAR+KB. Both exercise training interventions reduced HR peak during routines, *BLa*^*-*^, and slowed $\dot{V}O_{2}$ recovery presumably to facilitate PCr resynthesis following routines. Sports scientists should continue their search for innovative, feasible, and cost-effective protocols to improve the recovery patterns of female artistic gymnasts during competition.

### Pratical implications

The efficacy of claims credited with kettlebell exercise do not translate into improvements in cardiorespiratory and metabolic demands, and recovery kinetics during simulated competition of female artistic gymnastics. Possible explanations may relate to the selected load, swing cadence, duration, or lack of specificity of the REGULAR+KB protocol.

## Supporting information

S1 FileEffects of REGULAR and REGULAR+KB training on cardiorespiratory fitness and body composition.Data presented as mean (SD); Abbreviations: REGULAR, regular skill training; REGULAR+KB, regular skill training protocol + Kettlebell training; HR _max_, maximal heart rate, $\dot{V}O_{2\;\max}$ maximum oxygen uptake; $\dot{V}O_{2}$ at VT1 and $\dot{V}O_{2}$ at VT2 maximum oxygen uptake from the first and second ventilatory threshold. Post-hoc comparisons were performed for protocol-by-time interaction for each group, and for the main effect of group. * Indicates a significant difference from pre (*p* < 0.01). # Indicates a significant difference from REGULAR (*p* < 0.01).(DOCX)

S2 File Gymnastics scores before and after exercise training protocols. Data presented as mean (SD); Abbreviations: REGULAR, regular skill training; REGULAR+KB, regular skill training protocol + Kettlebell training. Post-hoc comparisons were performed for each group, and for the main effect of group.* Indicates a significant difference from pre (*p* < 0.01).
(DOCX)

S3 FilePhysiologic demand of female artistic gymnastic routines in selected indices of oxygen uptake and blood lactate.Abbreviations: $\dot{V}O_{2\mathrm{ext}}$_ext_, extrapolated maximal oxygen uptake during the routines; *BLa*^*-*^_*post1*,_ blood lactate 1 minute following each routine; *A*_*p*,_ amplitude of the $\dot{V}O_{2}$ in off-kinetics analysis; *HRR1*, heart rate 1 minute after each routine. *Indicates a significant difference from other routines (p < 0.05).(DOCX)

S1 Data (XLSX)
