# Supplementary material for: Effects of kettlebell swing training on cardiorespiratory and metabolic demand to a simulated competition in young female artistic gymnasts
Source: PLoS One. 2023 Apr 24;18(4):e0283228. doi: 10.1371/journal.pone.0283228 (PMC10124852; doi:10.1371/journal.pone.0283228)
Supplement: S1 File — Data presented as mean (SD); Abbreviations: REGULAR, regular skill training; REGULAR+KB, regular skill training protocol + Kettlebell training; HR max, maximal heart rate, V˙O2max maximum oxygen uptake; V˙O2 at VT1 and V˙O2 at VT2 maximum oxygen uptake from the first and second ventilatory threshold. Post-hoc comparisons were performed for protocol-by-time interaction for each group, and for the main effect of group. * Indicates a significant difference from pre (p < 0.01). # Indicates a significant difference from REGULAR (p < 0.01). (DOCX) [file pone.0283228.s001.docx]

| **Supplement 1.** Effects of REGULAR and REGULAR+KB training on oxygen uptake and heart rate during the cardiopulmonary exercise test. | | | | | | | | | | | |  |
| --- | --- | --- | --- | --- | --- | --- | --- | --- | --- | --- | --- | --- |
|  |  | | *Time* | | | *Training* | | | | *Interaction* | |  |
|  | REGULAR (n=9) | REGULAR+KB (n=9) | *p* | η ^2^ | | *p* | | η ^2^ | | *p* | η ^2^ |  |
| V̇O_2 max_ (L.min^-1^) |  |  |  | |  | |  | |  |  |  |  |
| Pre | 1.84 (0.78) | 1.53 (0.36) | **0.02** | | 0.34 | | 0.08 | | 0.19 | **0.008** | 0.42 |  |
| Post | 1.72 (0.70) | 1.92 (0.29) |  | |  | |  | |  |  |  |  |
| V̇O_2 max_ (mL.kg^-1^.min^-1^) |  |  |  | |  | |  | |  |  |  |  |
| Pre | 43.25 (3.30) | 36.00 (8.45) | **0.02** | 0.33 | | 0.34 | | 0.03 | | **0.009** | 0.37 |  |
| Post | 42.33 (4.05) | 44.77 (5.76) |  |  | |  | |  | |  |  |  |
| V̇O_2_ at VT1 (L.min^-1^) |  |  |  |  | |  | |  | |  |  |  |
| Pre | 1.05 (0.53) | 1.04 (0.37) | 0.32 | 0.06 | | 0.14 | | 0.13 | | 0.46 | 0.04 |  |
| Post | 1.25 (0.61) | 1.07 (0.39) |  |  | |  | |  | |  |  | |
| V̇O_2_ at VT1 (mL.kg^-1^.min^-1^) |  |  |  |  | |  | |  | |  |  |  |
| Pre | 24.99 (7.17) | 23.83 (6.94) | 0.22 | 0.05 | | 0.17 | | 0.06 | | 0.34 | 0.03 |  |
| Post | 30.82 (8.82) | 24.58 (7.71) |  |  | |  | |  | |  |  |  |
| V̇O_2_ at VT2 (L.min^-1^) |  |  |  |  | |  | |  | |  |  |  |
| Pre | 1.63 (0.65) | 1.41 (0.25)# | 0.06 | 0.22 | | 0.22 | | 0.09 | | **0.008** | 0.37 |  |
| Post | 1.54 (0.64) | 1.85 (0.35)* |  |  | |  | |  | |  |  |  |
| V̇O_2_ at VT2 (mL.kg^-1^.min^-1^) |  |  |  |  | |  | |  | |  |  |  |
| Pre | 38.68 (3.11) | 33.25 (6.65) | **0.02** | 0.30 | | 0.1 | | 0 | | **0.01** | 0.35 | |
| Post | 38.16 (6.88) | 43.09 (7.63)* |  |  | |  | |  | |  |  |  |
| HR _max_ (b.min^-1^) |  |  |  |  | |  | |  | |  |  |  |
| Pre | 156 (64) | 175 (10) | 0.32 | 0.06 | | 0.43 | | 0.04 | | 0.38 | 0.05 |  |
| Post | 176 (8) | 176 (9) |  |  | |  | |  | |  |  |  |
| Body fat (%) |  |  | 0.71 | 0 | | 0.14 | | 0.13 | | 0.69 | 0.01 |  |
| Pre | 13.30 (4.08) | 9.10 (6.53) |  |  | |  | |  | |  |  |  |
| Post | 12.89 (5.03) | 9.12 (6.62) |  |  | |  | |  | |  |  |  |
| Data presented as mean (SD); Abbreviations: REGULAR, regular skill training; REGULAR+KB, regular skill training protocol + Kettlebell training; HR _max_, maximal heart rate, V̇O_2_ _max_  maximum oxygen uptake; V̇O_2_ at VT1 and V̇O_2_ at VT2 maximum oxygen uptake from the first and second ventilatory threshold. Post-hoc comparisons were performed for protocol-by-time interaction for each group, and for the main effect of group.  * Indicates a significant difference from pre (*p* < 0.01).  # Indicates a significant difference from REGULAR (*p* < 0.01). | | | | | | | | | | | |  |
