# Supplementary material for: Effects of kettlebell swing training on cardiorespiratory and metabolic demand to a simulated competition in young female artistic gymnasts
Source: PLoS One. 2023 Apr 24;18(4):e0283228. doi: 10.1371/journal.pone.0283228 (PMC10124852; doi:10.1371/journal.pone.0283228)
Supplement: S2 File — Data presented as mean (SD); Abbreviations: REGULAR, regular skill training; REGULAR+KB, regular skill training protocol + Kettlebell training. Post-hoc comparisons were performed for each group, and for the main effect of group.* Indicates a significant difference from pre (p < 0.01). (DOCX) [file pone.0283228.s002.docx]

| **Supplement 2**. Gymnastics scores before and after exercise training protocols | | | | | | | | | | | | | | | | |
| --- | --- | --- | --- | --- | --- | --- | --- | --- | --- | --- | --- | --- | --- | --- | --- | --- |
|  | REGULAR | | | |  | REGULAR + KB | | | | *Time* | | *Protocol* | | *Interaction* | |  |
|  | Vault | Bars | Beam | Floor |  | Vault | Bars | Beam | Floor | *p* | *η^2^* | *p* | *η^2^* | *p* | *η^2^* |  |
| Pre | 11.1 (0.70) | 7.79 (1.15) | 10.22 (1.16) | 11.02 (0.90) |  | 11.12 (0.38) | 7.86 (1.05) | 9.93 (1.21) | 10.69 (0.77) | 0.64 | 0 | 0.42 | 0.04 | 0.46 | 0 |  |
| Post | 11.18 (0.69) | 8.09 (1.19) | 10.13 (1.26) | 10.99 (0.84) |  | 10.71 (0.46) | 8.06 (1.13) | 9.37 (1.30) | 10.3 (1.09) |  |  |  |  |  |  |  |
| Data presented as mean (SD); Abbreviations: REGULAR, regular skill training; REGULAR+KB, regular skill plus kettlebell training + skill training. Post-hoc comparisons were performed for each group, and for main effect of group.  * Indicates a significant difference from pre (*p* < 0.01). | | | | | | | | | | | | | | | | |
