# Supplementary material for: Effects of kettlebell swing training on cardiorespiratory and metabolic demand to a simulated competition in young female artistic gymnasts
Source: PLoS One. 2023 Apr 24;18(4):e0283228. doi: 10.1371/journal.pone.0283228 (PMC10124852; doi:10.1371/journal.pone.0283228)
Supplement: S3 File — Abbreviations: V˙O2extext, extrapolated maximal oxygen uptake during the routines; BLa-post1, blood lactate 1 minute following each routine; Ap, amplitude of the V˙O2 in off-kinetics analysis; HRR1, heart rate 1 minute after each routine. *Indicates a significant difference from other routines (p < 0.05). (DOCX) [file pone.0283228.s003.docx]

**Supplement 3** – Physiologic demand of female artistic gymnastic routines in selected indices of oxygen uptake and blood lactate.


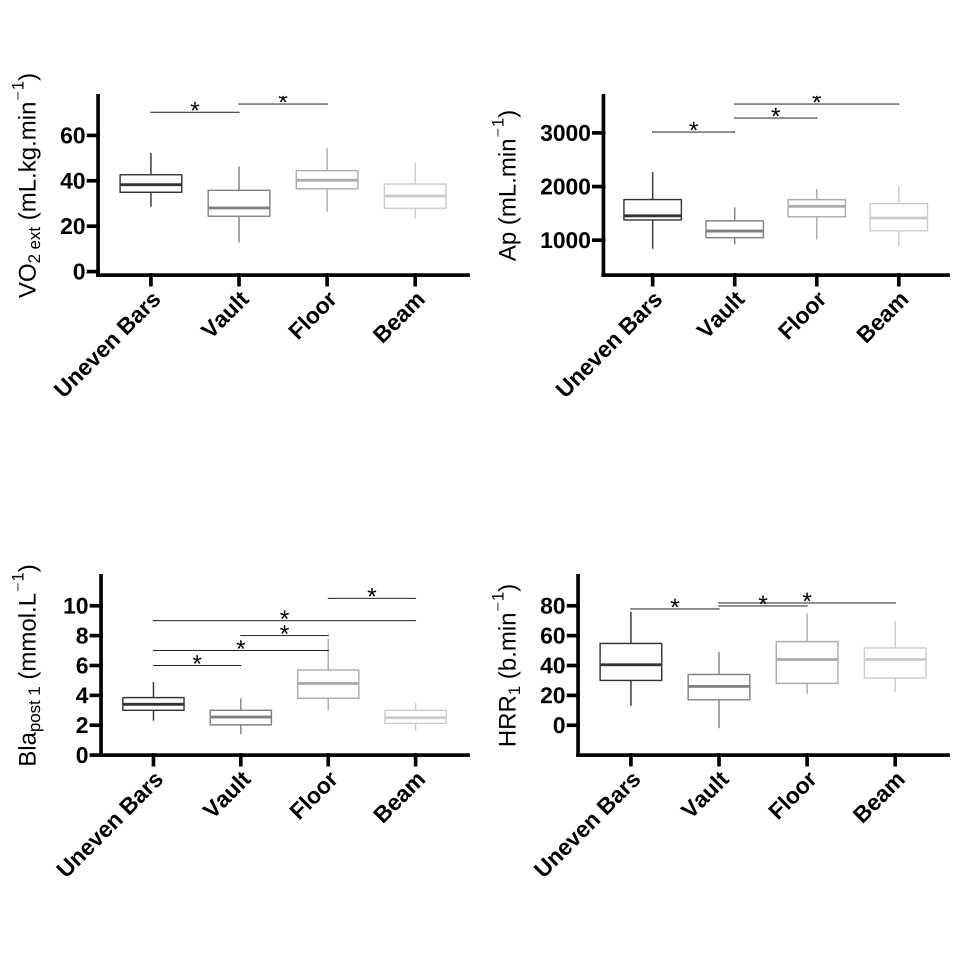


Abbreviations: V̇O_2 ext_, extrapolated maximal oxygen uptake during the routines; *BLa^-^_post1,_* blood lactate 1 minute following each routine; *A_p,_* amplitude of the V̇O_2_ in off-kinetics analysis; *HRR1,* heart rate 1 minute after each routine. *Indicates a significant difference from other routines (p < 0.05)
